# Choroidal Changes in Patients with Diabetic Retinopathy: A Retrospective Study

**DOI:** 10.3390/diagnostics14050537

**Published:** 2024-03-03

**Authors:** Shu-Yu Peng, Ta-Ching Chen, Yi-Ting Hsieh, Tzyy-Chang Ho, Chung-May Yang, Chang-Hao Yang

**Affiliations:** 1 Department of Ophthalmology, National Taiwan University Hospital, Taipei 10029, Taiwan; shuya30206@gmail.com (S.-Y.P.); tachingchen@gmail.com (T.-C.C.); ythyth@gmail.com (Y.-T.H.); hotchang@ntu.edu.tw (T.-C.H.); chungmay100@gmail.com (C.-M.Y.); 2 Department of Ophthalmology, College of Medicine, National Taiwan University, Taipei 10051, Taiwan

**Keywords:** diabetic retinopathy, diabetic choroidopathy, choroidal thickness, choroidal vascular index, LDL, OCT

## Abstract

This study aimed to investigate the characteristic choroidal changes in patients with diabetic retinopathy and identify factors affecting choroidal thickness (CTh), choroidal vascular index (CVI), and choriocapillaris flow. We retrospectively analyzed 79 eyes of 48 patients with diabetes between August 2021 and February 2022. We collected laboratory data, including HbA1c, serum creatinine, blood urea nitrogen, triglyceride, total cholesterol, high-density lipoprotein, and low-density lipoprotein (LDL) levels. Optical coherence tomography images of the foveal avascular zone, retinal vascular density, choroidal flow, retinal thickness, CTh, and CVI were analyzed. Possible determining factors affecting CTh, CVI, and choriocapillaris flow were analyzed using nonparametric multivariate analysis. LDL (*p* < 0.001) positively correlated with CTh, whereas CVI (*p* = 0.007) was negatively correlated with CTh in diabetic patients with diabetes. We also identified a negative correlation between choriocapillaris flow and deep parafoveal retinal vessel density in patients with low-grade diabetic retinopathy (DR), which diminished in those with more advanced DR. Our study provides further information on the changes in choroidal structure and blood flow in patients with diabetes.

## 1. Introduction

Diabetic retinopathy (DR) is a well-known microvascular complication of diabetes mellitus (DM). However, chronic hyperglycemia injures not only the retinal vessels but also the choroidal vessels. Because choroidal vessels supply blood to both the outer retina and choroid, they play an important role in the metabolism of the retina and choroid, especially in the foveal avascular zone (FAZ). Several studies have reported that choroidal changes in patients with diabetes may play an important role in the development of DR [1,2,3,4]. With advancements in optical coherence tomography (OCT), previous studies have demonstrated several choroidal changes associated with DR. These include changes in choroidal thickness (CTh) [5,6,7,8], choroidal vascular index (CVI) [9,10,11,12], and choriocapillaris flow. However, previous studies have reported conflicting results regarding diabetic choroidal changes.

Changes in CTh levels in DR have been described in several studies and have been associated with DR severity. Some studies found decreased CTh in more severe DR [5], whereas increased CTh in more severe DR was reported by other researchers [6]. Several other studies have described more complicated changes in CTh [7,8]. No single theory can perfectly resolve the complex and conflicting results of different studies. Additionally, other systemic and ophthalmic conditions can also exert influence on choroidal parameters, such as hypertension [13], hyperlipidemia, amblyopia [14], or myopia. Therefore, CTh in patients with diabetes may be influenced by factors that have not been previously identified, leading to conflicting results across studies.

The CVI has also been identified as an indicator of DR progression [15,16,17,18]. It is an OCT-based index, defined as the proportion of vascular areas to the total choroidal area. It may decrease with the progression of DR and may even precede its clinical manifestation. Furthermore, CVI has been proposed as a more stable method for evaluating choroidal health compared to measuring CTh alone. Despite this, there are limited studies analyzing the interaction between CVI and other choroidal changes in DR. We planned to clarify the relationship between them, which may provide further information on DR progression.

Choriocapillaris flow is decreased in patients with DR [9,10]. However, there are limited data on the relationship between choriocapillaris flow and other parameters such as retinal vascular density and CTh. A previous study reported that the choriocapillaris vascular perfusion density (VPD) was negatively correlated with the deep capillary plexus VPD of the retina [11]. The author postulated that the phenomenon may be caused by functional compensation to maintain the metabolic demand of the outer retina. However, that study only included patients with low-grade DR (no DR or mild nonproliferative DR). Therefore, a more comprehensive evaluation including patients with DR of all severities in a single study is required to confirm these findings.

Our study aimed to identify the relationships between CTh and CVI, choriocapillaris flow, and other possible factors in patients with diabetes.

## 2. Materials and Methods

### 2.1. Participants

This single-center retrospective study was conducted in compliance with our institutional review board’s regulations (National Taiwan University Hospital Research Ethics Committee) and the Declaration of Helsinki. The need for informed consent was waived by the ethics committee due to the retrospective study design. Medical records were retrieved from the electronic database of a tertiary referral center in Taiwan from 1 August 2021 to 1 February 2022 (the data were accessed for research purposes from 10 March 2022 to 1 July 2022). All data were fully anonymized after being processed. Patients with type 2 diabetes who were older than 20 years were included in this study. Patients were excluded if any of the following conditions were present: (1) other systemic diseases possibly affecting vascular architecture (systemic lupus erythematosus, coagulopathy); (2) other ocular diseases affecting CTh (central serous chorioretinopathy, age-related macular degeneration, axial length (AXL) longer than 26 mm, spherical equivalence (SE) of >−6 diopters) and retinal architecture (prominent macular edema, epiretinal membrane); (3) any retinal disease status post-panretinal photocoagulation; and (4) poor OCT image quality.

### 2.2. General Information and Laboratory Tests

We retrospectively collected basic information, including age, sex, underlying diseases, and ophthalmic history, from the electronic medical records. The following laboratory data were also retrieved: HbA1c, serum creatinine (Cre), blood urea nitrogen (BUN), eGFR, triglycerides (TG), total cholesterol (T-CHO), high-density lipoprotein cholesterol (HDL), and low-density lipoprotein cholesterol (LDL).

### 2.3. OCT Imaging

**Figure 1:**
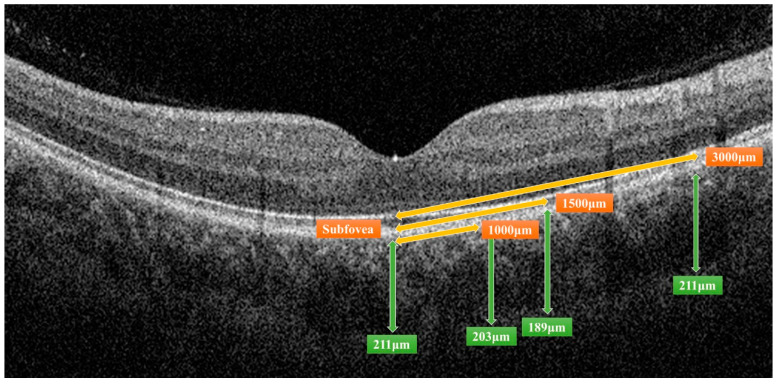
Measurement of choroidal thickness by the AngioVue Imaging System. The distance from the fovea, indicated by orange double-headed arrows, and the choroidal thickness of a certain distance, indicated by green double-headed arrows, were measured.

**Figure 2:**
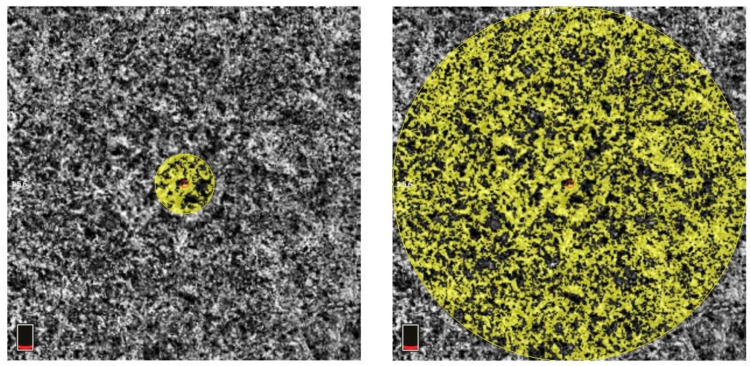
Measurement of central 500 µm (**left**) and 3000 µm (**right**) choriocapillaris flow. The area of measurement is indicated by the yellow circle. The red circle in the middle of the yellow circle is the center of the fovea. The boxes at the bottom left of both subfigures indicate that the scanning layer is located in the choroid layer.

**Figure 3:**
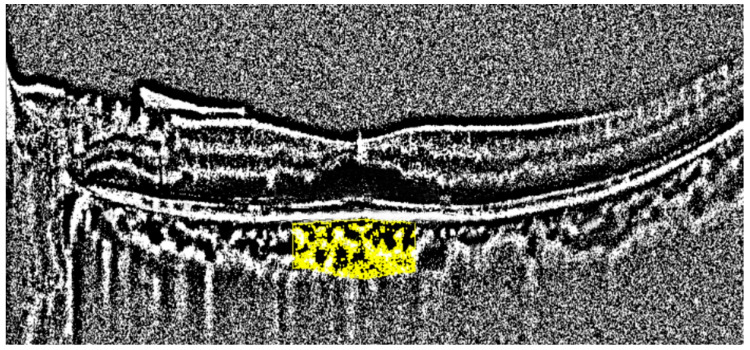
Measurement of the choroidal vascular index. The area of the analysis is marked in yellow.

All OCT images were retrieved using the AngioVue Imaging System (RTVue XR Avanti, Optovue Inc., Fremont, CA, USA). The OCT images were analyzed using a built-in automated layer segmentation program, which yielded the area of the FAZ, superficial parafoveal retinal vessel density (SVD), and deep parafoveal retinal vessel density (DVD). Further measurements of the OCT images were performed using built-in AngioVue software (RTVue RT-100, version 3.5; Optovue, Inc., Fremont, CA, USA). The CTh at the subfoveal area and 1000, 1500, and 3000 µm from the fovea in the nasal, temporal, superior, and inferior quadrants was measured manually by a single physician S.-Y.P. (Figure 1). The central 500 and 3000 µm choriocapillaris flow were calculated with the assistance of the AngioVue software (Figure 2). Choriocapillaris flow is presented in units of flow area (mm^2^). The CVI, defined as the ratio of the luminal area to the total choroidal area, was obtained using the image analysis algorithm proposed by Agrawal et al. [12] (Figure 3).

### 2.4. Statistical Analyses

Statistical analyses were performed using Microsoft Excel 2016 (Microsoft Inc., Redmond, WA, USA) and SPSS Version 22 (IBM Inc., Armonk, NY, USA). To identify the significant factors associated with CTh/CVI/choriocapillaris flow, we performed a univariate regression analysis to assess the association between CTh/CVI/choriocapillaris flow and other parameters. Clinical significance was defined as *p* < 0.05. These significant factors were then fitted to a multivariate generalized estimating equation (GEE) to identify the factors determining CTh/CVI/choriocapillaris flow in patients with DR.

## 3. Results

**Figure 4:**
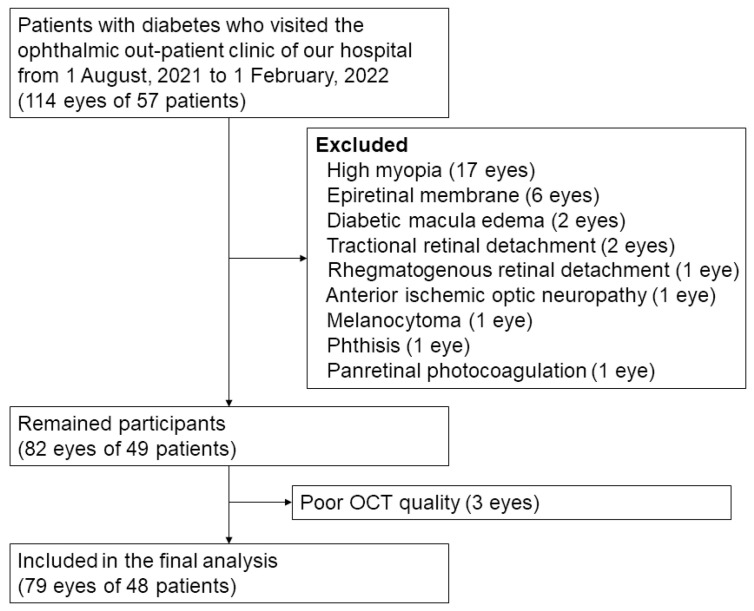
Flow diagram of study participants.

This study included 79 eyes of 48 patients with diabetes (Figure 4). The demographic and clinical characteristics of the participants are shown in Table 1. The average age of the patients was 66.7 ± 10.8 years. Among them, 23 (47.9%) were men and 25 (52.1%) were women. The average HbA1c was 7.7 ± 1.9%. The patients were classified as having no DR (23 eyes, 29.1%); mild NPDR (21 eyes, 26.6%); moderate NPDR (15 eyes, 19.0%); severe NPDR (12 eyes, 15.2%); and proliferative DR (PDR) (eight eyes, 10.1%) according to the ETDRS classification.

### 3.1. Clinical Characteristics of DR of Different Severities

**Figure 5:**
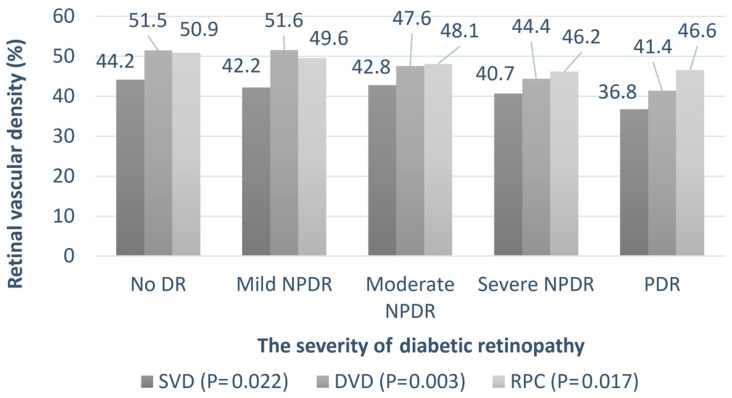
The relationship between the retinal vessel densities and severity of diabetic retinopathy. Univariate regression analysis is performed to assess the association between DR severity and retinal vessel densities. The *p*-values are shown in parentheses. SVD: superficial parafoveal retinal vessel density, DVD: deep parafoveal retinal vessel density, RPC: radial peripapillary capillary density.

**Figure 6:**
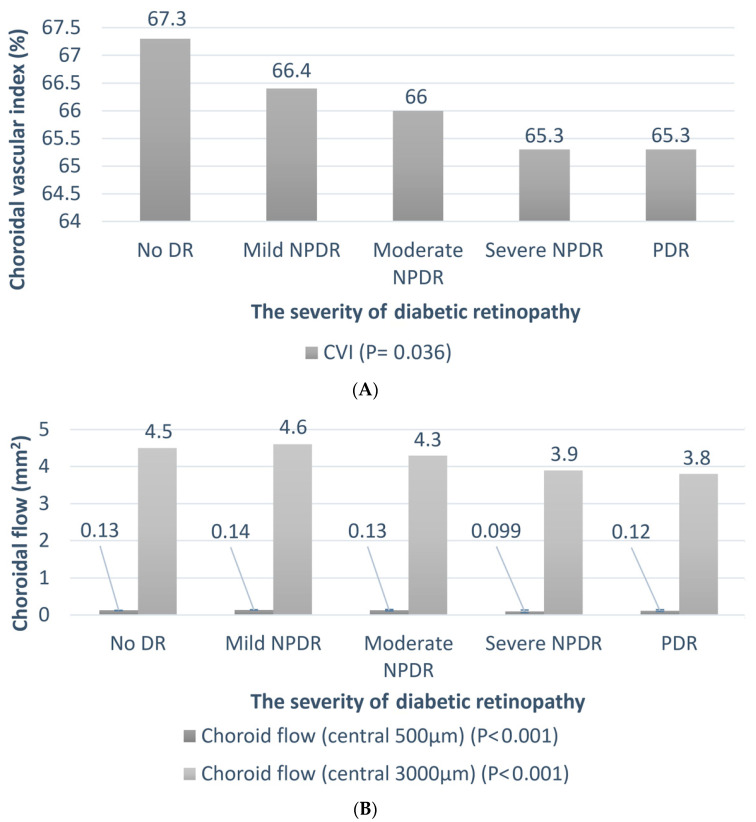
The relationship between the choroidal vessel and severity of diabetic retinopathy. Univariate regression analysis is performed to assess the association between DR severity and CVI/choroidal flow. The *p*-values are shown in parentheses. (**A**) The trend of the choroidal vascular index (CVI); (**B**) the trend of central 500 and 3000 µm choroidal flow.

Table 2 summarizes the clinical features of the patients with DR of different severities. Univariate regression analysis was performed to assess the association between DR severity and other parameters. As expected, the HbA1c levels were higher in patients with severe DR (*p* < 0.001). The trend of the change in the chorioretinal vessels is shown in Figure 5 and Figure 6A,B. The average SVD (*p* = 0.022), average DVD (*p* = 0.003), average radial peripapillary capillary density (*p* = 0.017), and CVI (*p* = 0.036) were significantly lower in the more severe stages of DR. The choroid flow area in the central 500 and 3000 µm was also decreased in more advanced DR (*p* < 0.001). The details are shown in Table S1.

### 3.2. Choroidal Thickness (CTh)

The CTh values in the DR for different severities are listed in Table 3. The average CTh was thicker in the mild NPDR group, thinner in the moderate NPDR group, and thicker in the severe NPDR and PDR groups. However, the trend was not statistically significant. The significance of the difference between the no DR and other DR groups is shown in Table 3. The details are shown in Table S2.

To identify the significant factors associated with CTh, univariate regression was employed to assess the association between CTh and other parameters. The results are summarized in Table S3. To simplify the calculation, CTh values at 1000 and 3000 µm from the fovea in the nasal, temporal, superior, and inferior quadrants were averaged at each distance in this analysis (average CTh at 1000 µm = the average CTh at 1000 µm from the fovea in the nasal, temporal, superior, and inferior quadrants). According to the univariate regression analysis, age, AXL, and CVI were significantly negatively correlated with CTh. In contrast, the SE, LDL, and T-CHO levels were significantly and positively correlated with CTh.

These significant factors were then fitted to a multivariate GEE to identify the factors determining CTh levels in patients with DR. SE was not included in multivariate analysis because it was strongly associated with AXL. Similarly, since LDL is part of T-CHO, only LDL was included in the multivariate analysis. Table 4 presents the results of the GEE analysis. In this analysis, CTh in the subfoveal region and the average CTh at all locations had a significant positive association with LDL. Age and CVI were negatively associated with CTh at all locations. Sex and AXL were not associated with CTh in any location.

### 3.3. Choroidal Vascular Index (CVI)

Univariate regression analysis was performed to assess the association between CVI and other parameters. The results are summarized in Table S4. AXL, DR severity, BUN, and CTh at all locations showed significant negative correlations with CVI. Notably, LDL levels did not correlate with the CVI.

These significant factors were further analyzed using GEE (Table 5). AXL and BUN levels were not significant in multivariate analysis. Age, DR severity, and CTh at all locations were significantly negatively correlated with the CVI. As LDL correlated with CTh in the previous analysis, we added LDL to the GEE analysis to validate our findings. The results confirmed that the correlations of other factors remained unchanged, and LDL was not correlated with the CVI.

### 3.4. Choriocapillaris Flow

#### 3.4.1. All DR Patients

We performed univariate regression of choriocapillaris flow within the central 500 and 3000 µm areas for all patients with DR (Table S5). Unsurprisingly, HbA1c levels were negatively correlated with choriocapillaris flow. The eGFR, SVD, and DVD were positively correlated with choriocapillaris flow within either the central 500 or 3000 µm area.

We simplified the calculation of SVD and DVD using only the average SVD and DVD to perform the GEE calculations (e.g., average SVD = average of superior, inferior, nasal, and temporal SVD). After fitting those significant factors into the GEE, eGFR and average SVD had a significant positive correlation with central 500 µm choriocapillaris flow. However, the average DVD was not significant in this analysis (Table 6).

#### 3.4.2. Low-Grade DR Patients

Next, we focused on the patients with low-grade DR (no DR and mild NPDR). Univariate regression of choriocapillaris flow within the central 500 µm and 3000 µm from the fovea was analyzed (Table S6). As creatinine levels were significantly correlated with choriocapillaris flow in patients with low-grade DR, this parameter was added to the GEE analysis. The results are summarized in Table 7. SVD still had the trend of being positively correlated with central 500 µm choriocapillaris flow. In contrast, DVD was significantly negatively correlated with central 500 µm choriocapillaris flow.

## 4. Discussion

Choroidal changes in patients with DR are complicated and may be influenced by several factors. In the present study, we identified LDL levels and CVI as important factors affecting CTh in patients with DR. Our study also confirmed a negative correlation between the choriocapillaris and deep retinal capillary plexus in patients with mild DR, which was not observed in patients with advanced DR.

Changes in the CTh levels in patients with DR have been explored in several previous studies. However, these studies reported conflicting results [4,5,6,7,8,19]. For instance, decreased CTh correlated with increased severity of DR, according to Horvath et al. (96 eyes of 48 patients with diabetes; average HbA1c = 7.64 ± 1.11%; Hungary) [5]. However, Endo et al. [8] reported that CTh decreased in mild and moderate NPDR and paradoxically increased in severe NPDR and PDR (228 eyes of 134 patients with diabetes; average HbA1c = 7.5 ± 1.3% and 10.0 ± 2.5% in DM-treated and untreated groups; Japan). In contrast, Wang et al. [7]. demonstrated that CTh increased in mild NPDR; however, it decreased in more severe DR stages (1347 patients with diabetes; average HbA1c= 6.9 ± 1.4%; China). Hamadneh et al. reviewed several studies focusing on the relationship between CTh changes and different DR stages [4]. They stated that the increase in CTh in DR may be due to the upregulated secretion of intraocular vascular endothelial growth factor (VEGF). Because VEGF leads to hyperpermeability of choroidal vessels, it may increase CTh levels in patients with DR. In contrast, decreased CTh may be explained by decreased choriocapillaris perfusion and progressive ischemia of the choroid. Owing to the complexity of choroidal changes, we postulated that changes in CTh in patients with DR may be affected by factors other than DR severity.

In our study, LDL levels significantly and positively correlated with CTh levels in patients with DR. Previous studies focusing on the relationship between CTh and LDL levels have reported inconsistent results. Cougnard-Gregoire et al. [20] reported no association between CTh and LDL levels in a population-based study (867 eyes of 440 subjects; France). In contrast, Wei et al. [21] reported that subfoveal CTh was positively correlated with cholesterol levels in univariate analysis in another population-based study (3233 subjects; China). However, this significance was diminished in the multivariate analysis. Another study by Yazgan et al. [22], which focused on patients with prediabetes, did not show an association between LDL and CTh (53 eyes of 53 patients with prediabetes; Turkey). Wong et al. [23] compared hypercholesterolemia patients with healthy controls, determining that subfoveal CTh and CTh 1 mm from the fovea were significantly higher in the hypercholesterolemia group than those in the control group (322 eyes of 161 subjects; Hong Kong, China). To our knowledge, this is the first study to focus on patients with DM and to confirm that LDL is positively correlated with CTh at multiple locations using multivariate analysis. These findings can be explained by an animal experiment conducted by Salazar et al. [24]. A thickened choroid with atherosclerotic changes was observed in the hypercholesterolemic rabbits. Human eyes may exhibit similar atherosclerotic changes when LDL levels are elevated [25], which leads to increased CTh. Another possible mechanism is the increased inflammation caused by LDL and atherosclerosis [26]. Inflammation may cause vascular leakage and choroidal swelling, which manifests as a thickened choroid.

Moreover, in this study, the CVI significantly negatively correlated with CTh in patients with DR. The CVI is an indicator of DR progression [15,16,17,18], and our study confirmed this association. However, few studies have discussed the relationship between CVI and CTh. In two studies conducted by Kim et al. [27,28], the CVI was positively correlated with subfoveal CTh, whereas CTh at other locations did not show a significant correlation (230 eyes and 161 eyes, respectively; Korea). Multivariate linear regression rather than nonparametric multivariate regression was used in their study. In contrast, our study showed that the CVI was negatively correlated with CTh. Initially, we thought that this finding might be another phenomenon caused by chronic hypercholesterolemia, according to another study conducted by Kim et al. [29]. They reported an association between atherosclerosis and decreased CVI. We postulated that as LDL is deposited in the choroidal vessels, atherosclerosis leads to increased CTh, while choroidal vessel diameter decreases simultaneously. Decreased choroidal vessel diameter can result in decreased CVI. However, further GEE analysis did not reveal any correlation between the CVI and LDL levels. Further studies are required to determine the exact pathophysiology of the negative correlation between CTh levels and CVI in patients with DR.

The relationship between the choriocapillaris and retinal vascular plexus is also not well understood. Most of these studies included patients with DR of all severities and reported that both retinal capillary plexus and choriocapillaris densities decreased with more severe DR [9,10,30,31,32]. According to a report by Lupidi et al. [11], choriocapillaris VPD is negatively correlated with deep capillary plexus VPD of the retina in low-grade DR (29 eyes of 29 patients with diabetes, 20 eyes of 20 healthy controls; average HbA1c = 7.6 ± 0.5%; Italy). This phenomenon may be caused by functional compensation in patients with DR. If the deep retinal capillary plexus is compromised, the perfusion of the choriocapillaris may increase to maintain the metabolic demand of the outer retina. In contrast, if choriocapillaris flow is insufficient, the deep retinal capillary plexus may increase its flow similarly. This compensatory mechanism is disrupted in more advanced DR owing to further capillary damage. Our study further confirmed these findings in patients with low-grade DR. Because we included patients with DR at all stages in a single study, our study is the first comprehensive study to show that choriocapillaris flow is negatively correlated with retinal deep vascular density in low-grade DR, and this correlation diminishes with increasing DR severity.

This study had some limitations. First, owing to its retrospective design, some laboratory data were missing. Second, our OCT machine cannot automatically calculate choroidal thickness, and manual measurements may be suboptimal. Third, the PDR group in our study was younger than the other groups and the sample size was relatively small, which may have affected the results. Fourth, our study did not include the duration of DM, which may have significantly affected the choroidal thickness. Despite these limitations, our study provides important information on the choroidal changes in patients with DM.

In conclusion, this study identified LDL level and CVI as important factors affecting CTh in patients with DR. LDL positively correlated with CTh, whereas CVI negatively correlated with CTh in multiple locations in patients with DM. We also confirmed a negative correlation between the choriocapillaris flow and DVD in patients with mild DR, which was not observed in patients with more advanced DR. Our study provides further information on choroidal changes in patients with DR.

## Figures and Tables

**Table 1:** Demographic and clinical characteristics of the participants.

Parameter	Number
Patients (no.)	48
Eyes (no.)	79
Age (years) (mean ± SD)	66.7 ± 10.8
Sex (male/female)	23:25
Side (right/left)	40:39
AXL (mm)	23.8 ± 1.1 (excluded > 26 mm)
BCVA ^a^ (LogMAR)	0.32 ± 0.33
HbA1c (%)	7.7 ± 1.9
Diabetic retinopathy classification (no. of eyes)	
No DR	23
Mild NPDR	21
Moderate NPDR	15
Severe NDPR	12
PDR	8

^a^ BCVA: best-corrected visual acuity.

**Table 2:** Clinical features in different DR severities.

Parameter	No DR	Mild NPDR	Moderate NPDR	Severe NPDR	PDR	Significance
Eyes (no.)	23	21	15	12	8	-
Age (years) (mean ± SD)	69.2 ± 11.7	66.5 ± 11.5	66.9 ± 8.1	64.6 ± 6.1	54.9 ± 15.9	*0.076*
AXL (mm)	23.7 ± 0.87	23.5 ± 1.3	23.4 ± 0.89	24.4 ± 1.1	25.48	*0.562*
BCVA (LogMAR)	0.26 ± 0.26	0.17 ± 0.16	0.24 ± 0.17	0.69 ± 0.52	0.47 ± 0.36	*0.017*
Lab data						
HbA1c (%)	6.8 ± 0.94	7.3 ± 0.42	7.3 ± 0.83	8.1 ± 1.9	10.85 ± 3.1	*<0.001*
Cre (mg/dL)	1.4 ± 1.7	0.93 ± 0.29	2.1 ± 2.8	1.2 ± 0.67	1.4 ± 1.0	*0.684*
BUN (mg/dL)	35.6 ± 15.7	20.2 ± 1.8	32.4 ± 29.5	22.4 ± 5.4	26.3	*0.438*
eGFR (mL/min/1.73 m^2^)	73.2 ± 28.0	80.4 ± 19.6	67.8 ± 34.8	69.9 ± 33.3	69.6 ± 37.5	*0.675*
TG (mg/dL)	116.1 ± 42.5	96.2 ± 39.5	94.2 ± 25.5	139.6 ± 67.5	150.3 ± 90.6	*0.439*
HDL (mg/dL)	59.7 ± 14.9	61.4 ± 14.6	66	64	72	*0.099*
LDL (mg/dL)	91.1 ± 20.5	107.1 ± 29.4	76.6 ± 2.9	88.9 ± 23.6	153.3 ± 23.7	*0.334*
T-CHO (mg/dL)	161.2 ± 34.4	182.7 ± 27.7	150.0 ± 3.5	157.4 ± 22.5	251	*0.367*
GCC ^a^ (average) (μm)	93.7 ± 9.2	89.1 ± 20.6	93.6 ± 14.8	120.8 ± 15.1	123 ± 32.3	*<0.001*
RNFL ^b^ (average) (μm)	101.2 ± 23.2	103.4 ± 13.3	91.4 ± 15.3	110.8 ± 23.9	122.1 ± 21.3	*0.076*

Univariate regression analysis is performed to assess the association between DR severity and other parameters. ^a^ GCC: ganglion cell complex, ^b^ RNFL: retinal nerve fiber layer.

**Table 3:** Choroid thickness in different diabetic retinopathy severities.

Location of CTh	No DR *n* = 23	Mild NPDR *n* = 21	Moderate NPDR *n* = 15	Severe NPDR *n* = 12	PDR *n* = 8
Subfoveal	215.4 ± 95.2	234.1 ± 99.2	193.9 ± 70.5	253.9 ± 81.8	297.7 ± 81.0
*p*-value	-	*0.406*	*0.406*	*0.052*	*0.076*
1000 μm (average)	199.0 ± 85.3	223.3 ± 94.8	180.0 ± 65.0	235.0 ± 71.1	260.0 ± 66.5
*p*-value	-	*0.285*	*0.525*	*0.052*	*0.085*
1500 μm (average)	192.9 ± 78.5	209.7 ± 84.5	174.6 ± 61.0	226.3 ± 64.1	247.3 ± 65.9
*p*-value	-	*0.428*	*0.489*	*0.050*	*0.085*
3000 μm (average)	169.7 ± 67.8	182.1 ± 60.5	160.2 ± 48.5	196.9 ± 46.4	235.0 ± 40.8
*p*-value	-	*0.285*	*0.765*	*0.014*	*0.024*

The choroidal thickness for each severity level of DR is compared with the choroidal thickness in patients without DR (Wilcoxon rank-sum test). Unit: μm.

**Table 4:** Generalized estimating equation results of choroidal thickness.

Parameter	CTh Location (Average) *	Beta Estimations	Significance
Age (years)	Subfoveal	−4.054	*0.003*
	1000 μm	−3.454	*0.004*
	3000 μm	−2.307	*0.007*
Sex (male, female)	Subfoveal	−12.359	*0.597*
	1000 μm	−12.330	*0.544*
	3000 μm	−14.588	*0.384*
AXL (mm)	Subfoveal	−0.287	*0.797*
	1000 μm	−0.658	*0.568*
	3000 μm	−0.318	*0.699*
LDL (mg/dL)	Subfoveal	1.210	*<0.001*
	1000 μm	0.996	*<0.001*
	3000 μm	0.618	*0.003*
CVI (%)	Subfoveal	−13.247	*0.007*
	1000 μm	−13.296	*0.007*
	3000 μm	−8.297	*0.019*

* The CTh values at different distances from the fovea were analyzed separately. The results of the generalized estimation equation were similar for different CTh locations.

**Table 5:** Generalized estimating equation results of choroidal vascular index.

Parameter	Beta Estimations	Significance
Age (years) *	−0.315	0.003
AXL (mm) *	0.094	0.057
DR severity *	−1.525	0.010
BUN (mg/dL) *	0.026	0.320
Choroid thickness (average)		
Subfoveal	−0.009	0.038
1000 μm ^#^	−0.009	0.050
1500 μm ^#^	−0.012	0.035
3000 μm ^#^	−0.019	0.020

* Data on age, AXL, DR severity, and BUN shown in this table were calculated when subfoveal CTh was included in the GEE. ^#^ Different CTh locations were added to the analysis instead of subfoveal CTh, and the significance of other parameters remained grossly unchanged.

**Table 6:** Generalized estimating equation of choriocapillaris flow in all patients with DR.

Parameter	Choroid Flow Area (Diameter) *	Beta Estimations	Significance
Age (years)	500 μm	9.06 × 10^−5^	*0.640*
	3000 μm	−0.006	*0.467*
HbA1 (%)	500 μm	−3.73 × 10^−4^	*0.893*
	3000 μm	−0.148	*<0.001*
eGFR (mL/min/1.73 m^2^)	500 μm	2.23 × 10^−4^	*0.011*
	3000 μm	0.001	*0.755*
SVD (average) (%)	500 μm	0.001	*0.001*
	3000 μm	−0.009	*0.314*
DVD (average) (%)	500 μm	−4.16 × 10^−4^	*0.433*
	3000 μm	0.008	*0.105*

* The central 500 and 3000 µm choriocapillaris flows are analyzed separately.

**Table 7:** Generalized estimating equation of choriocapillaris flow in low-grade DR.

Parameter	Choroid Flow Area (Diameter) *	Beta Estimations	Significance
Age (years)	500 μm	−1.80 × 10^−4^	*0.441*
	3000 μm	−0.013	*0.060*
HbA1 (%)	500 μm	−0.005	*0.095*
	3000 μm	−0.189	*0.023*
eGFR (mL/min/1.73 m^2^)	500 μm	−1.38 × 10^−4^	*0.385*
	3000 μm	−0.008	*0.001*
Cre (mg/dL)	500 μm	−0.008	*0.003*
	3000 μm	−0.167	*<0.001*
SVD (average) (%)	500 μm	0.001	*0.051 (Trend)*
	3000 μm	−0.033	*0.001*
DVD (average) (%)	500 μm	−0.002	*0.018*
	3000 μm	−0.004	*0.462*

* The central 500 and 3000 µm choriocapillaris flows are analyzed separately.

## Data Availability

Data are available on request due to restrictions: The data presented in this study are available on request from the corresponding author. The data are not publicly available due to privacy.

## Supplementary Materials

The following supporting information can be downloaded at: https://www.mdpi.com/article/10.3390/diagnostics14050537/s1, Table S1: Detailed clinical characteristics of patients with diabetic retinopathy (DR) of different severities; Table S2: Choroid thickness and choroidal flow in diabetic retinopathy of different severities; Table S3: Univariate regression results for choroidal thickness (CTh); Table S4: Univariate regression results for choroidal vascular index (CVI); Table S5: Univariate regression results for choriocapillaris flow for all patients with diabetic retinopathy (DR); Table S6: Univariate regression of choriocapillaris flow in low-grade patients with diabetic retinopathy (DR).
