# Supplementary material for: Choroidal Changes in Patients with Diabetic Retinopathy: A Retrospective Study
Source: Diagnostics (Basel). 2024 Mar 3;14(5):537. doi: 10.3390/diagnostics14050537 (PMC10930972; doi:10.3390/diagnostics14050537)
Supplement: Supplementary file 1 [file diagnostics-14-00537-s001.zip › diagnostics-2889018-supplementary.pdf]

Table S1. Detailed clinical characteristics of patients with diabetic retinopathy (DR) of different severities

Parameter	No DR	Mild NPDR	Moderate NPDR	Severe NPDR	PDR
**Eyes (no.)**	23	21	15	12	8
**Age (years) (mean ± SD)**	69.2±11.7	66.5±11.5	66.9±8.1	64.6±6.1	54.9±15.9
**AXL (mm)**	23.7±0.87	23.5±1.3	23.4±0.89	24.4±1.1	25.48
**BCVA** (LogMAR)	0.26±0.26	0.17±0.16	0.24±0.17	0.69±0.52	0.47±0.36
**Laboratory data**					
**HbA1c** (%)	6.8±0.94	7.3±0.42	7.3±0.83	8.1±1.9	10.85±3.1
**Cre** (mg/dL)	1.4±1.7	0.93±0.29	2.1±2.8	1.2±0.67	1.4±1.0
**BUN** (mg/dL)	35.6±15.7	20.2±1.8	32.4±29.5	22.4±5.4	26.3
**eGFR** (mL/min/1.73 m^2)	73.2±28.0	80.4±19.6	67.8±34.8	69.9±33.3	69.6±37.5
**TG** (mg/dL)	116.1±42.5	96.2±39.5	94.2±25.5	139.6±67.5	150.3±90.6
**HDL** (mg/dL)	59.7±14.9	61.4±14.6	66	64	72
**LDL** (mg/dL)	91.1±20.5	107.1±29.4	76.6±2.9	88.9±23.6	153.3±23.7
**T-CHO** (mg/dL)	161.2±34.4	182.7±27.7	150.0±3.5	157.4±22.5	251
**Retinal vascular change**					
**SVD (average)** (%)	44.2±6.5	42.2±4.1	42.8±4.3	40.7±7.4	36.8±6.2
**DVD (average)** (%)	51.5±6.5	51.6±4.9	47.6±4.7	44.4±5.6	41.4±4.2
**RPC (average)** (%)	50.9±2.9	49.6±4.9	48.1±4.5	46.2±4.0	46.6±4.7
**FAZ (mm)**	0.32±0.13	0.35±0.15	0.47±0.20	0.34±0.08	0.28±0.14
**Choroid flow** (mm^2)					
**Central 500 µm**	0.13±0.0019	0.14±0.027	0.13±0.020	0.099±0.036	0.12±0.026
**Central 3000 µm**	4.5±0.46	4.6±0.27	4.3±0.21	3.9±0.29	3.8±0.44
**CVI** (%)	67.3±2.7	66.4±2.5	66.0±3.8	65.3±1.9	65.3±2.3
**GCC (average)** (µm)	93.7±9.2	89.1±20.6	93.6±14.8	120.8±15.1	123±32.3
**RNFL (average)** (µm)	101.2±23.2	103.4±13.3	91.4±15.3	110.8±23.9	122.1±21.3

Table S2. Choroid thickness and choroidal flow in diabetic retinopathy of different severities

	**No DR**<br><br>N=23	**Mild NPDR**<br><br>N=21	**Moderate NPDR**<br>N=15	**Severe NPDR**<br>N=12	**PDR**<br><br>N=8
**Choroidal thickness**					
**Subfoveal**	215.4±95.2	234.1±99.2	193.9±70.5	253.9±81.8	297.7±81.0
**Superior**					
1000 µm	209.7±92.8	240.0±104.4	190.6±74.6	254.9±75.9	270.5±72.7
1500 µm	204.7±86.5	233±96.6	184.2±67.3	257.4±78.1	266.7±59.1
3000 µm	201.3±70.6	227.3±74.7	187.5±60.4	238±76.6	250.2±33.8
**Inferior**					
1000 µm	193.1±89.0	216.3±97.9	173.9±66.8	238.4±84.6	258.3±68.1
1500 µm	195.1±81.5	207.2±94.9	168.8±62.8	240.1±86.7	245.7±57.3
3000 µm	174.7±76.6	170.4±71.3	153.4±51.1	223.5±73.7	242.0±35.0
**Nasal**					
1000 µm	189.2±86.7	209.1±100.9	173.5±75.2	224.3±76.2	246.2±65.5
1500 µm	172.6±93.3	184.3±89.1	162.9±73.4	190.7±58.6	226.3±80.3
3000 µm	125.6±77.7	133.9±73.1	128.7±60.7	130.8±49.8	192.8±52.2
**Temporal**					
1000 µm	204.1±95.5	227.8±93.2	182.2±64.4	222.5±59.5	265.0±74.5
1500 µm	199.3±84.9	214.0±77.3	182.5±70.6	217.0±53.0	250.5±80.1
3000 µm	177.4±71.3	196.8±59.7	171.0±59.1	195.4±31.1	254.8±66.4
**Average**					
1000 µm	199.0±85.3	223.3±94.8	180.0±65.0	235.0±71.1	260.0±66.5
1500 µm	192.9±78.5	209.7±84.5	174.6±61.0	226.3±64.1	247.3±65.9
3000 µm	169.7±67.8	182.1±60.5	160.2±48.5	196.9±46.4	235.0±40.8
**Choroid flow** (mm^2)					
Central 500 µm	0.13±0.0019	0.14±0.027	0.13±0.020	0.099±0.036	0.12±0.026
Central 3000 µm	4.5±0.46	4.6±0.27	4.3±0.21	3.9±0.29	3.8±0.44
Unit: µm					

Table S3. Univariate regression analysis of the choroidal thickness (CTh)

Parameter	Location of CTh (Average)	Standardized coefficients	Significance
**Age (years)**	Subfoveal	-0.429	<0.001
	1000 μm	-0.425	<0.001
	1500 μm	-0.422	<0.001
	3000 μm	-0.479	<0.001
**Sex (male, female)**	N/A	-	-
**BCVA (LogMAR)**	N/A	-	-
**AXL (mm)**	3000 μm	-0.273	0.020
**Spherical equivalence (diopter)**	Subfoveal	0.282	0.016
	1000 μm	0.251	0.032
	1500 μm	0.233	0.047
**DR severity**	N/A	-	-
**HbA1 (%)**	N/A	-	-
**Cre (mg/dL)**	N/A	-	-
**BUN (mg/dL)**	N/A	-	-
**eGFR (mL/min/1.73 m^2)**	N/A	-	-
**TG (mg/dL)**	N/A	-	-
**HDL (mg/dL)**	N/A	-	-
**LDL (mg/dL)**	Subfoveal	0.464	0.001
	1000 μm	0.440	0.001
	1500 μm	0.440	0.001
	3000 μm	0.444	0.001
**T-CHO (mg/dL)**	Subfoveal	0.477	0.001
	1000 μm	0.436	0.003
	1500 μm	0.439	0.003
	3000 μm	0.385	0.009
**SVD (%)**	N/A	-	-
**DVD (%)**	N/A	-	-
**Choroid flow (mm^2)**	N/A	-	-
**CVI (%)**	Subfoveal	-0.387	0.002
	1000 μm	-0.409	0.001
	1500 μm	-0.389	0.001
	3000 μm	-0.406	0.001

The CTh values at different distances from the fovea are analyzed separately. “N/A” denotes that the choroidal thickness at all locations is not correlated with the parameter.

Table S4. Univariate regression analysis of the choroidal vascular index (CVI).

Parameter	Standardized coefficients	Significance
**Age (years)**	-	-
**Sex (male, female)**	-	-
**BCVA (LogMAR)**	-	-
**AXL (mm)**	-0.0272	0.030
**Spherical equivalence (Diopter)**	-	-
**DR severity**	-0.0551	0.040
**HbA1 (%)**	-	-
**Cre (mg/dL)**	-	-
**BUN (mg/dL)**	-0.053	0.031
**eGFR (mL/min/1.73 m^2)**	-	-
**TG (mg/dL)**	-	-
**HDL (mg/dL)**	-	-
**LDL (mg/dL)**	-	-
**T-CHO (mg/dL)**	-	-
**SVD (%)**		
Foveal	-	-
Superior	-	-
Inferior	-	-
Nasal	-	-
Temporal	0.25	0.050
Average	-	-
**DVD (%)**		
Foveal	-	-
Superior	-	-
Inferior	0.247	0.053
Nasal	-	-
Temporal	0.239	0.061
Average	-	-
**Choroid thickness (average)**		
Subfoveal	-0.387	0.002

1000 µm	-0.409	0.001
1500 µm	-0.389	0.001
3000 µm	-0.406	0.001

"-"

denotes that the CVI is not correlated with the parameter.

Table S5. Univariate regression results for choriocapillaris flow in all patients with diabetic retinopathy (DR)

Parameter	Choroid flow area (diameter)	Standardized coefficients	Significance
**Age (years)**	N/A	-	-
**BCVA (LogMAR)**	500 μm	0.406	<0.001
**AXL (mm)**	N/A	-	-
**Spherical equivalence**	3000 μm		
**HbA1 (%)**	500 μm<br>3000 μm	-0.234<br>-0.549	0.060<br>0.000
**Cre (mg/dL)**	N/A	-	-
**BUN (mg/dL)**	N/A	-	-
**eGFR (mL/min/1.73 m^2)**	500 μm	0.371	0.003
**TG/HDL/LDL/T-CHO (mg/dL)**	N/A	-	-
**SVD (%)**			
Foveal	3000 μm	-0.306	0.007
Superior	500 μm	0.262	0.022
Inferior	500 μm	0.254	0.026
Nasal	500 μm	0.308	0.007
Temporal	500 μm<br>3000 μm	0.293<br>0.250	0.010<br>0.028
Average	500 μm	0.317	0.005
**DVD (%)**			
Foveal	N/A		
Superior	3000 μm	0.416	<0.001
Inferior	3000 μm	0.294	0.010
Nasal	3000 μm	0.443	<0.001
Temporal	3000 μm	0.431	<0.001
Average	3000 μm	0.332	0.030
**CVI (%)**	N/A		
**Choroid thickness** (μm)	N/A	-	-

The central 500 and 3000 μm choriocapillaris flows are analyzed separately. "N/A" denotes that the choriocapillaris flow within the central 500 or 3000 μm is not correlated with the parameter.

Table S6. Univariate regression analysis of the choriocapillaris flow in patients with low-grade diabetic retinopathy (DR).

**Parameter**	**Choroid flow area (diameter)**	**Standardized Coefficients**	**Significance**
**Age (years)**	N./A	-	-
**BCVA (LogMAR)**	N/A	-	-
**AXL (mm)**	N/A	-	-
**Spherical equivalence (diopter)**	N/A	-	-
**HbA1 (%)**	N/A	-	-
**Cre (mg/dL)**	3000 μm	-0.325	0.043
**BUN (mg/dL)**	N/A	-	-
**eGFR (mL/min/1.73 m^2)**	N/A	-	-
**TG/HDL/LDL/T-CHO (mg/dL)**	N/A	--	-
**SVD (%)**			
Foveal	3000 μm	-0.396	0.008
Superior	N/A	-	-
Inferior	N/A	-	-
Nasal	N/A	-	-
Temporal	N/A	-	-
Average	N/A	-	-
**DVD (%)**			
Foveal	N/A		
Superior	500 μm	-0.460	0.002
Inferior	N/A	-	-
Nasal	500 μm	-0.321	0.036
Temporal	N/A	-	-
Average	500 μm (Trend)	-0.287	0.059
**CVI (%)**	N/A		
**Choroid thickness (μm)**	N/A	-	-

The central 500 and central 3000 μm choriocapillaris flows are analyzed separately. "N/A" denotes that the choriocapillaris flow within the central 500 or 3000 μm is not correlated with the parameter.
